# *Drosophila* SNAP-29 Is an Essential SNARE That Binds Multiple Proteins Involved in Membrane Traffic

**DOI:** 10.1371/journal.pone.0091471

**Published:** 2014-03-13

**Authors:** Hao Xu, Mahmood Mohtashami, Bryan Stewart, Gabrielle Boulianne, William S. Trimble

**Affiliations:** 1 Department of Biological Sciences, University of Southern Mississippi, Hattiesburg, Mississippi, United States of America; 2 Department of Immunology, Sunnybrook Research Institute, Toronto, Ontario, Canada; 3 Department of Biology, University of Toronto Mississauga, Mississauga, Ontario, Canada; 4 Developmental and Stem Cell Biology Program, Hospital for Sick Children, and Department of Molecular Genetics, University of Toronto, Toronto, Ontario, Canada; 5 Cell Biology Program, Hospital for Sick Children, and Department of Biochemistry, University of Toronto, Toronto, Ontario, Canada; Medical College of Georgia, United States of America

## Abstract

Each membrane fusion event along the secretory and endocytic pathways requires a specific set of SNAREs to assemble into a 4-helical coiled-coil, the so-called trans-SNARE complex. Although most SNAREs contribute one helix to the trans-SNARE complex, members of the SNAP-25 family contribute two helixes. We report the characterization of the *Drosophila* homologue of SNAP-29 (dSNAP-29), which is expressed throughout development. Unlike the other SNAP-25 like proteins in fruit fly (i.e., dSNAP-25 and dSNAP-24), which form SDS-resistant SNARE complexes with their cognate SNAREs, dSNAP-29 does not participate in any SDS-resistant complexes, despite its interaction with dsyntaxin1 and dsyntaxin16 in vitro. Immunofluorescence studies indicated that dSNAP-29 is distributed in various tissues, locating in small intracellular puncta and on the plasma membrane, where it associates with EH domain-containing proteins implicated in the endocytic pathway. Overexpression and RNAi studies suggested that dSNAP-29 mediates an essential process in *Drosophila* development.

## Introduction

Vesicular fusion requires SNAREs (SNAP receptors) located on the transport vesicle (e.g., VAMP/synatobrevin) to interact with cognate SNAREs from the target membrane (e.g., syntaxin and SNAP-25) [1], [2]. Such interaction leads to the formation of a stable 4-helical trans-SNARE complex, whose subsequent disassembly after fusion depends on the actions of an ATPase NSF (N-ethyl-maleimide-sensitive factor) and its cofactor SNAP (soluble NSF attachment protein) [3]. The notion that each trafficking event requires a unique set of SNAREs [4] has been supported by the identification of many SNAREs within the syntaxin and VAMP/synaptobrevin families, with rather unique but sometimes overlapping distribution patterns along the secretory and endocytic pathways. SNAP-25 like proteins are unique in that each contributes two helices to the trans-SNARE complex. The virtually completed human, worm and fly genomes all seem to suggest just 3 or at most 4 members in the SNAP-25 family: SNAP-25, SNAP-23, SNAP-29 [5], and SNAP-47 [6].

SNAP-25 is expressed almost exclusively in the brain and required mainly at the plasma membrane of the nerve terminals [7], [8]. Botulinum neurotoxin A and E cleave the C-terminal domain of SNAP-25, causing a partial inhibition of secretion in the neuronal system [9]. Consistent with this, overexpression of a truncated form of SNAP-25, which lacks the last nine residues at the C-terminus, led to a reduction of exocytosis in chromaffin cells [10]. SNAP-23 appears to be the functional isoform of SNAP-25 in non-neuronal tissues [11], [12]. Like SNAP-25, SNAP-23 localizes mainly to the plasma membrane and is involved in exocytosis from both polarized and nonpolarized cell types [13], [14], [15], [16], [17], [18]. Overexpression of SNAP-24, a putative *Drosophila* orthologue of SNAP-23, rescues the synaptic transmission defect and lethality caused by the loss of SNAP-25 in the fruit fly [19]. Similarly, ubiquitously-distributed SNAP-47 is able to substitute for SNAP-25 in SNARE complex formation with the neuronal SNARE syntaxin 1 and VAMP/synaptobrevin 2 [6]. Unlike SNAP-25, SNAP-47 is enriched in synaptic vesicle fractions of the neuron rather than in the plasma membrane [6] and is required in postsynaptic rather than presynaptic exocytosis [20].

In contrast to SNAP-23, SNAP-25, and SNAP-47, which are mainly to support membrane fusion at the cell surface, SNAP-29 appears to participate in a wide range of fusion events in the cell. Mammalian SNAP-29 is distributed on multiple membranes including Golgi, endosomes, and lysosomes where it can interact with multiple syntaxins [21] [22], [23]. In mast cells, SNAP-29 is recruited to *E. coli*-containing phagosomes to facilitate phagocytosis and the subsequent killing of the bacteria [24]. In fibroblast cells derived from CEDNIK (Cerebral Dysgenesis, Neuropathy, Ichthyosis and Keratoderma) patients, the loss of functional SNAP-29 impaired endocytic recycling and cell motility without affecting secretion [25]. Depletion of SNAP-29 in C. elegans blocks secretion in both polarized and nonpolarized cells [26], which leads to cytokinesis defects and sterility [27]. In addition, SNAP-29 inhibits synaptic transmission when introduced into cultured presynaptic superior cervical ganglion neurons, by preventing SNARE complex disassembly [28]. More recently, SNAP-29 has been functionally linked to starvation-induced autophagy in both man and fruit fly [29], [30]. These studies suggest that SNAP-29 mediated fusion is required in diverse biological processes in eukaryotes.

To distinguish the unique function of each SNAP-25 related molecule in membrane traffic, binding partners beyond the SNARE super-family are being identified and studied. To-date, mammalian SNAP-25 has been shown to interact with SNIP (SNAP-25-interacting protein), Hrs (hepatocyte growth factor-regulated tyrosine kinase substrate), and intersectin. SNIP is a brain-specific cytoskeleton-associating protein and may serve as a linker to connect SNAP-25 with the submembranous cytoskeleton [31]. Hrs prefers to localize to the endosomal membrane which generates PI(3)P [32] and has recently been assigned a clear role in the maturation of multi-vesicular body (MVB) [33], [34], [35]. However, Hrs also appears to be implicated in calcium-regulated secretion [36], [37]. Intersectin contains two Eps 15 homology (EH) domains and five SH3 domains, which allow the protein to associate with the endocytic machinery [38]. The observation that intersectin binds SNAP-25 and SNAP-23 has led to the postulation that it may function to couple the endocytic membrane traffic to exocytosis. Interestingly, mammalian SNAP-29 has also been found to interact with a EH domain-containing protein EHD1 [39] [25], although the physiological significance of the interaction has yet to be examined.

Here we report the characterization of the SNAP-29 orthologue in *Drosophila*, which was initially identified via a yeast 2-hybrid screen using dSNAP as bait. dSNAP-29 is ubiquitously expressed throughout the fly life cycle, interacting with multiple proteins including dSNAP, dsyntaxin1, dsyntaxin 16, dEHD1, and DAP160. Overexpression and RNAi studies show that the function of dSNAP-29 is essential in *Drosophila*.

## Materials and Methods

### Cloning and subcloning of *Drosophila SNAP-29*

A partial cDNA of *Drosophila SNAP-29* was cloned by a yeast 2-hybrid screen using dSNAP as bait [40]. A blast search of Flybase allowed the identification of an apparent full-length EST clone LD17127, which was obtained from Research Genetics. Full-length *dSNAP29* or that lacking the C-terminal 25 amino acids (*dSNAP29ΔC*)was amplified by PCR with primers: 5′-GAATTCCATGGCCCATAACTACCTGCAGC and 5′-AGATCTCGAGTCACTTCTTCAGAAGCTTGCTC, or 5′-AGATCTCGAGTCAGTTATCCAGCAATTCATTTTGCG, and then subcloned into the *Sma* I site of pBluescript SK^+^ vector (Stratagen) for sequencing

*dSNAP-29* was then subcloned into pGEX-KG to generate GST-dSNAP-29 for binding assays, into pQE-30 to generate His-dSNAP-29 for antibody production, and into pRmHa-3-myc for transient transfection. *dSNAP-29* and *dSNAP-29ΔC* were each inserted into the polycloning site of pUAST for microinjection.

The sequences of SNAP-25-related proteins were obtained from GenBank™. Multiple sequence alignments were performed using the ClustalW program (using default parameters), which also generates a phylogenetic tree.

### Antibody preparation and immunostaining

Peptide CKQNKDMSKLLKK (the N-terminal C was added to faciliate crosslinking) was synthesized (University of Toronto) and coupled to maleimide-activated carrier protein keyhole limpet hemocyanin (Pierce), which was then used to immunize rabbits. To purify the antibody, the peptide was immobilized onto SulfoLink Coupling Gel (Pierce), incubated overnight with the antiserum and then washed with 2 M NaCl in PBS, 0.1 M boric acid (pH 9.1), and PBS (pH 4.5) consecutively. The purified antibody was eluted with 20 mM glycine (pH 2.5) and neutralized immediately with 100 mM Tris (pH 8.5).

To prepare antibody against the full-length protein, *dSNAP-29* was subcloned into the pQE-30 vector (Qiagen) and expressed in *E. coli* strain BSJ72. His-dSNAP-29 was purified on Ni^+^ beads (Qiagen) for rabbit immunization. Around 150 μg of His-dSNAP-29 were run on an SDS-polyacrylamide gel, transferred onto nitrocellulose filter, and then isolated for antibody purification.

Immunofluorescence studies of salivary glands, imaginal discs, and transfected S2 cells were carried out as described ([41]. On some occasions, S2 cells were fixed with cold methanol for 5 min on ice. Overnight collections of *Oregan R* embryos were dechorinated with 50% bleach for 2 min, shaked in a scintillation vail with 50% n-heptane and 50% fixation solution (4% paraformaldehyde in 0.1 M Na_3_PO_4_, pH 7.2) for 30 min, washed with methanol 3 times, dehydrated with ethanol (for storage at −80 °C), and then rehydrated before staining. Images of salivary glands, S2 cells embryos were captured by a Zeiss LSM510 confocal microscope.

### Fractionation, binding assays, immunoprecipitation and western blotting

To determine the membrane association of dSNAP-29, the membrane fraction of an adult fly lysate was separated from the soluble fraction, and treated as described [41]. Subcellular fractionation was described in [42].

Binding assays measuring the direct interaction between recombinant dSNAP-29 and dSNAP were performed with 200 ng of GST-dSNAP-29 (see [41] for details). To identify potential binding partners of dSNAP-29, approximately 200 million Schneider S2 cells were lysed in 0.5 mL of PLC or NP40 lysis buffer. Supernatant supplemented with 0.1% gelatin and 0.1% BSA was pre-cleared with glutatione beads at 4 °C for 1 hr, before incubation with 0.5 mg of GST-dSNAP-29, GST-dSNAP-25, or GST (each coupled with 120 μL of glutathione beads). Following extensive washes with 50 mM HEPES (pH 7.5), 150 mM NaCl, and 2 mM EDTA, agarose beads were boiled with 120 μL of 2×SDS sample buffer which was then subject to SDS-PAGE and western blot analysis. Binding assays measuring the direct interaction between recombinant dSNAP-29 and dSNAP were performed with 200 ng of GST-dSNAP-29 (see [41] for details).

For immunoprecipitation studies, overnight collections of *Oregan R* embryos (1.5 mL) were homogenized in 1 mL of homogenization buffer (HKA, 1 mM PMSF, 2 mM benzimidine, 2 μg/mL leupeptin, 2 μg/mL pepstatin, 5 mM EDTA). Following centrifugation at 600 g for 10 min, the supernatant was incubated with 4% Triton X-100 for 1 hr at 4 °C and then subject to a second centrifugation at 13,000 g for 10 min. About 0.5 mL of the supernatant was then incubated with 2.5 μg of affinity purified anti-SNAP-29 or pre-immune serum that had been cross-linked to protein A beads. Following a 2 hr incubation at 4 °C, the beads were washed extensively with HKA, 150 mM NaCl, 2% Triton X-100 before extraction with 45 μL of 2× SDS sample buffer containing 10 mM NEM. 10 μL were loaded each time for SDS-PAGE and subsequent western blot analysis.

To examine SDS-resistant SNARE complexes, *comt^TP7^* or wild-type adult flies were incubated at 37 °C for 10 min, after which the heads were collected and homogenized directly in 1% SDS lysis buffer. Half of the amount of each sample was boiled while the other half was not in order to preserve the complexes.

To measure the relative protein levels on the western blot, the chemi-luminescence from each band was captured directly by the FluorChem™ Imaging System (Alpha Innotech).

### Double strand RNA interference

For RNAi on cultured S2 cells, d*SNAP29* cDNA was amplified from pQE-30-dSNAP29 by PCR using primers carrying the minimal T7 promoter: 5′-TTAATACGACTCACTATAGGGAGAGGATCGCATCACCATCACC and 5′-TTAATACGACTCACTATAGGGAGATCTATCAACAGGAGTCCAAGC. The PCR product was purified from agarose gel (QIAEX II gel extraction kit, Qiagen), and then used to synthesize RNA with an in vitro transcription kit, MEGAscript (Ambion). The dsRNA was annealed by incubation at 65 °C for 30 min followed by gradual cooling to room temperature. For each RNAi experiment, 15 μg of dsRNA were incubated with 1 million S2 cells for 5 to 6 days [43].

To introduce dsRNA into *Drosophila*, *dSNAP-29* cDNA from pGEX-dSNAP-29 was inserted into pUAST [44] via *Xho* I and *Xba* I sites, such that the full-length cDNA is inverted. *dSNAP-29ΔC* was subsequently subcloned into *EcoR* I and *Bgl* II sites upstream of the inverted full-length cDNA. This gave rise to a tail-to-tail repeat of *dSNAP-29ΔC* (775 bp) located downstream of the GAL4-dependent upstream activating-sequence (UAS), which was used for microinjection to create transgenic lines.

### Fly stocks and genetic studies

Stocks were maintained at room temperature on standard cornmeal agar medium unless otherwise indicated. Visible markers and balancer chromosomes have been previously described [45]. Transgenic flies *UAS-dSNAP-29*, *UAS- dSNAP-29ΔC*, and *UAS-dSNAP-29^RNAi^* were created by standard P-element-mediated transformation [46]. Individual UAS lines were crossed with GAL4 lines to activate the ectopic expression of various forms of *dSNAP-29*.

## Results

### Temporal and spatial distribution of dSNAP-29/ubisnap

**Figure 1:**
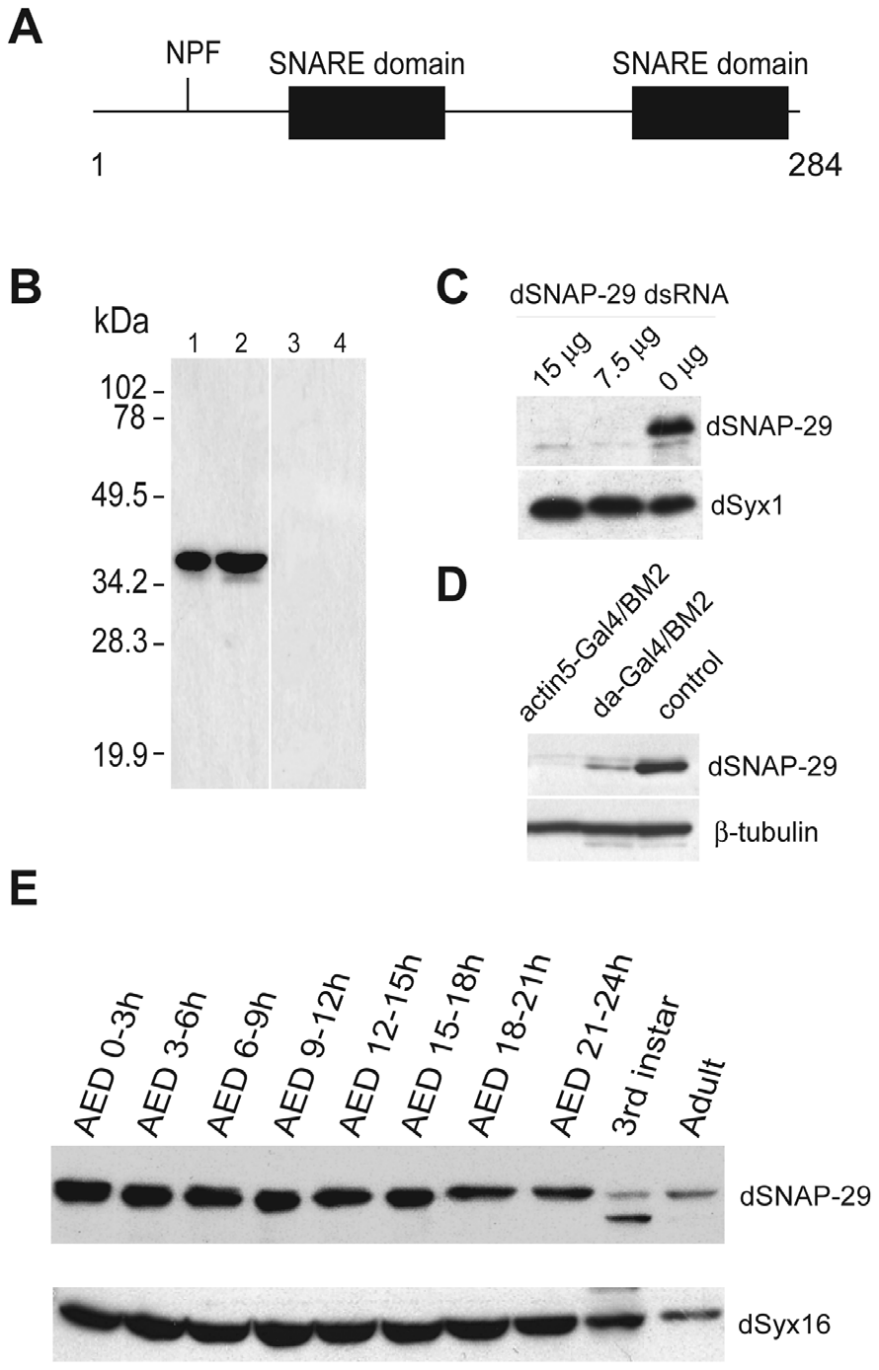
dSNAP-29 is expressed throughout the fly life cycle. A) Domain structure of dSNAP-29. B). Affinity-purified anti-dSNAP29 preincubated with (lanes 3 and 4) or without (lanes 1 and 2) the peptide was used to blot 10 μg (lanes 1 and 3) and 20 μg (lanes 2 and 4) of total proteins extracted from adult flies. C) S2 cells were treated with dsRNA of *dSNAP-29* for 5 days after which the cells were lysed and immunoblotted by anti-dSNAP29 and anti-dSyx1. D) Transgenic line UAS-dSNAP29^RNAi-BM2^ was crossed with GAL4 lines to allow ectopic expression of dsRNA of *dSNAP-29*. Inhibition of protein synthesis was demonstrated by western blotting analysis. E) Embryos after deposition (AED) were collected and allowed to develop for indicated period of time. 20 μg of proteins from each of the embryonic collections, larvae, and adults were subject to SDS-PAGE and western blotting analysis.

*Drosophila* SNAP-29 (CG11173) was identified in a yeast 2-hybrid screen for proteins that interact with *Drosophila* SNAP [40]. Like the other two SNAP-25 like proteins in *Drosophila (i.e., dSNAP-24 and dSNAP-25)*, dSNAP-29 possesses two terminal alpha-helical SNARE domains that are predicted to form coiled-coils (Fig. 1A). However, dSNAP-29 lacks a cysteine cluster and a QPXR(V/I) motif in between the SNARE domains, which are required for anchoring SNAP-25/23 isoforms to the plasma membrane [47], [48], [49]. In contrast, dSNAP-29 has a unique N-terminal NPF motif (Fig. 1A), which is involved in the interaction with Eps15 homology (EH) domains, a feature shared by all known SNAP-29 isoforms (reviewed by [50]).

To distinguish dSNAP-29 from other endogenous fly proteins, antibody against a 12 amino-acid peptide at the C-terminus of the protein was raised in rabbit and then affinity-purified. The peptide antibody recognizes a 35 kDa band from the adult fly lysate (Fig. 1B), slightly above the predicted molecular mass of the protein (32 kDa). The band disappears if the antibody is pre-incubated with the peptide (Fig. 1B, lanes 3 and 4). To further confirm the specificity of the antibody, we applied double-stranded RNA of dSNAP-29 to cultured Schneider 2 (S2) cells, which results in the loss of the immuno-detection of the 35 kDa band from cell lysates (Fig. 1C). The treatment had no effect on the detection of dsyntaxin1 (Fig. 1C). Similarly, in transgenic flies where double-stranded RNA of dSNAP-29 (UAS-dSNAP-29^RNAi^) was expressed under the control of ubiquitous Gal4 drivers (i.e., actin5-GAL4 or daughterless-Gal4), the level and subsequent detection of dSNAP-29 were selectively reduced (Fig. 1D). We thus conclude that the peptide antibody we generated is specific for dSNAP-29.

**Figure 2:**
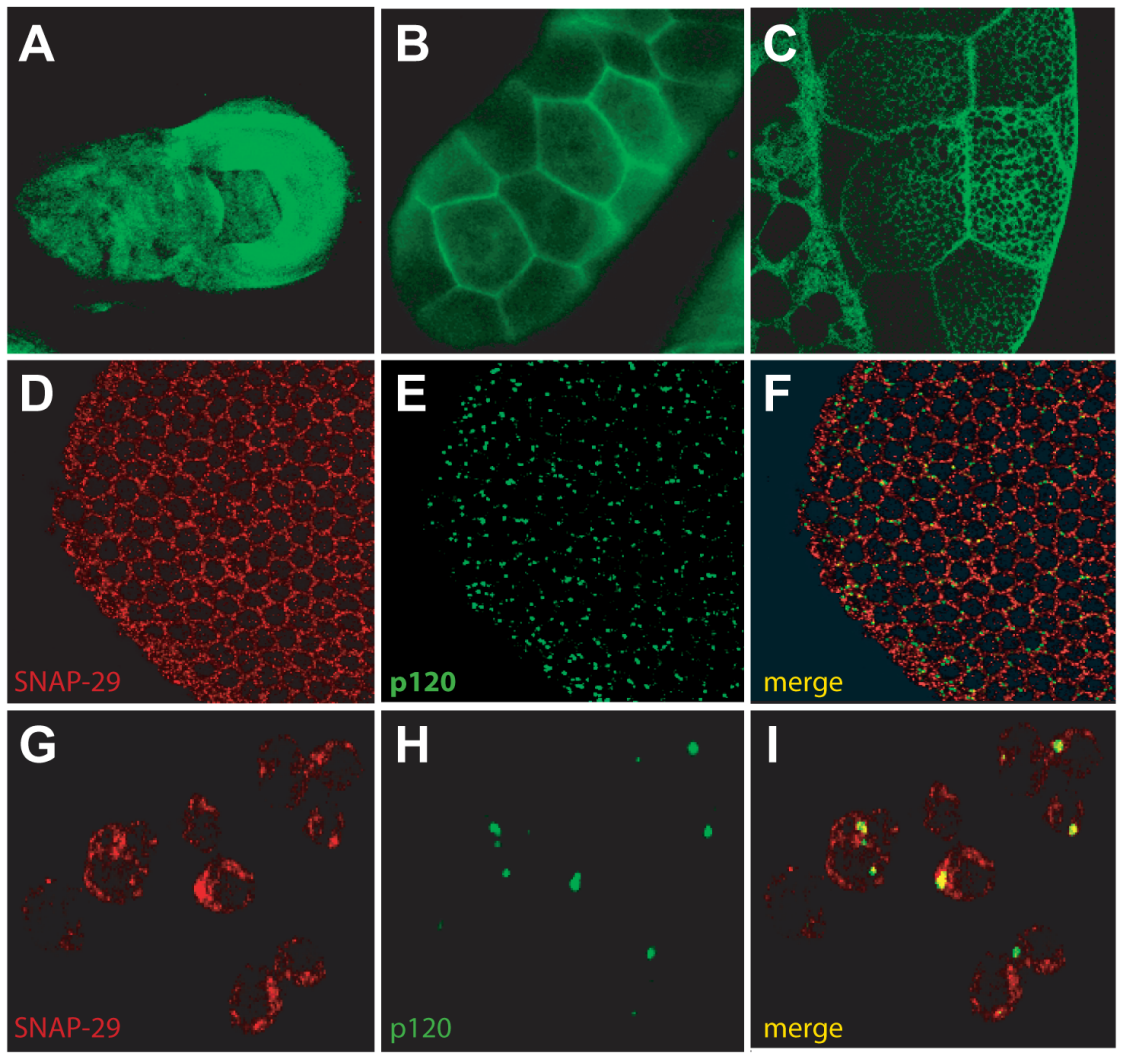
dSNAP-29 is widely distributed in *Drosophila*. Using indirect immuofluorescence, dSNAP29 was detected in wing disc A), salivary gland cells B) and C), early embryos D), and S2 cells G). The samples in D) and G) were co-stained with anti-p120 as shown in E) and H). G) and J) are merged images.

To examine the temporal expression of dSNAP-29, embryos from *Oregon R* were collected every three hours after embryo deposition (AED) and allowed to develop for up to 24 hours. Embryos, 3^rd^ instar larva, and adults were then lysed and equal amounts of total protein were separated by SDS-PAGE and immunoblotted. As shown in Fig. 1E (top lane), dSNAP-29 is expressed in all developmental stages examined, albeit with more abundant levels in early embryos. This is slightly different from dsyntaxin16, which appears to be more evenly expressed throughout the embryonic stages (Fig. 1E, bottom lane). The relative levels of both dSNAP-29 and dsyntaxin16 are reduced in larval and adult stages, probably reflecting the increased production of other proteins in these stages. We went on to determine the spatial localization of dSNAP-29 at various developmental stages. Immunocytochemistry performed on overnight embryonic collections suggests that dSNAP-29 is ubiquitously expressed in embryos (data not shown). Using indirect immunofluorescence, we detected in early embryos, a strong perinuclear staining pattern that appears to be different from that of the Golgi (Fig. 2D to F). However, the narrow cytoplasmic space between the nucleus and the plasma membrane precludes a more defined localization of dSNAP-29 in these cells. In cultured S2 cells, the distribution patten of dSNAP-29 is also different from that of the Golgi marker p120 (Fig. 2 compare G to H), but there appear to be some limited colocalization between the two, suggesting that dSNAP-29 might mediate membrane traffic to and/or from the Golgi. In 3^rd^ instar larva, dSNAP-29 has been found to distribute widely: from imaginal discs to salivary glands (Fig. 2A to C). A close inspection of salivary gland cells with confocal microscopy suggests that dSNAP-29 is not just in the cytoplasm but also on the plasma membrane. This is in contrast to dSNAP-25, which is not detected in salivary glands, and dSNAP-24, which is predominantly localized onto the secretory granules [51]. Because of the ubiquitous distribution of dSNAP-29, we had initially named the gene Ubisnap (usnp).

**Figure 3:**
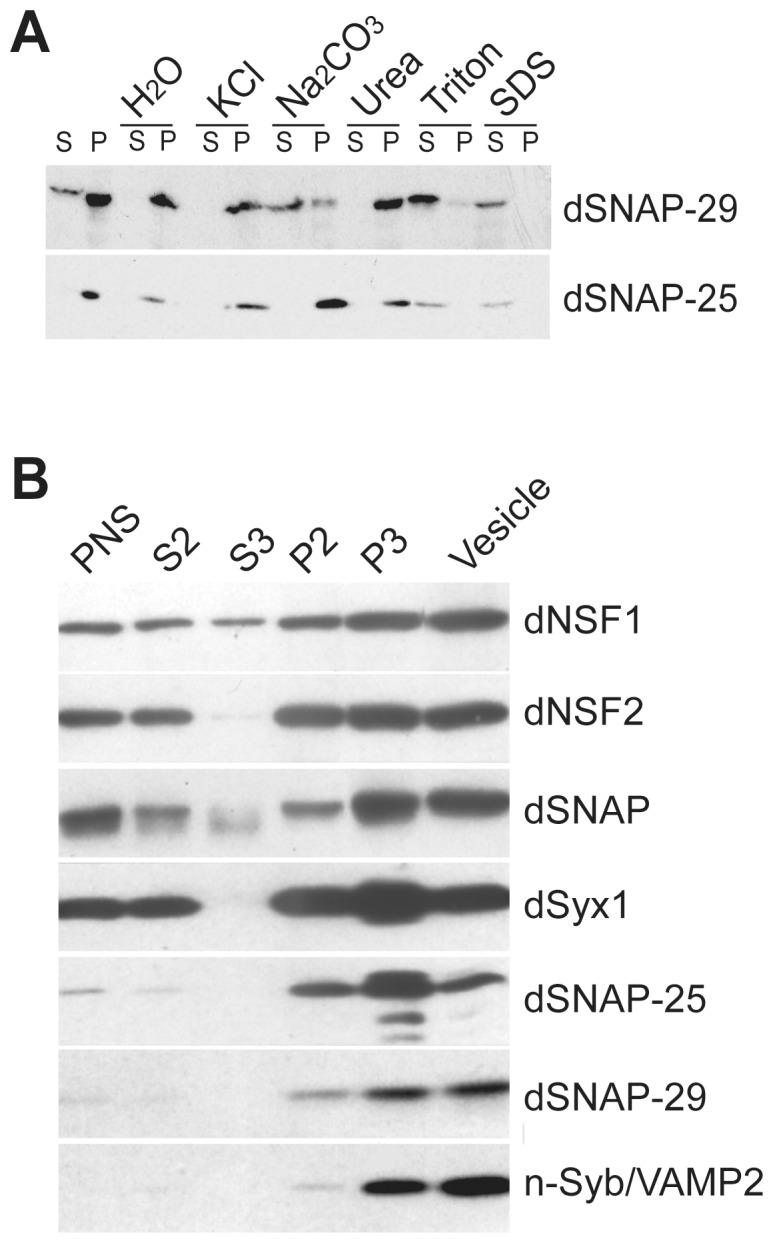
Fractionation studies of dSNAP-29. In A), crude membrane fraction from homogenized *Oregon R* adults was treated with H_2_O, KCl, Na_2_CO_3_, Urea, Triton X-100, or SDS and then centrifuged to separate the soluble from the insoluble. In B), a post nuclear supernatant (PNS) from fly head homogenate was fractionated into the pellet (P2) and the supernatant (S2) fractions by centrifugation. The S2 fraction was then centrifuged on a discontinuous sucrose gradient to separate cytosol (S3), crude vesicles and the plasma membrane (P3). 10 μg of total protein from each fraction were subject to SDS-PAGE and blotted with various antibodies indicated on the right.

The detection of dSNAP-29 on the plasma membrane of the salivary gland cells persuaded us to investigate the nature of its association with the membrane. Adult fly lysate was separated into soluble and membrane fractions by centrifugation. Subsequent SDS-PAGE and western blotting analysis showed that although dSNAP-29 was predominant in the membrane fraction, a small yet significant portion was also present in the soluble fraction. This is different from dSNAP-25, which was exclusively distributed in the membrane fraction (Fig. 3A). This difference between the two proteins was more evident after the membrane fraction was treated with KCl, Na_2_CO_3_ (high pH), urea, Triton X-100 and SDS respectively. While both dSNAP-29 and dSNAP-25 resisted the extraction by KCl and urea, dSNAP-29 appeared to be more sensitive to Na_2_CO_3_, suggesting that dSNAP-29 may require hydrophobic interactions to bind its membrane receptors. Similar observations have been reported for mammalian SNAP-29s. Considering the fact that SNAP-29 isoforms lack the cysteine cluster and the QPXR(V/I) motif, it is likely that they use an alternative but less stringent mechanism to associate with the membrane.

To further characterize the subcellular localization of dSNAP-29, the post-nuclear supernatant (PNS) from fly head homogenate was fractionated into the pellet (P2) and the supernatant (S2) fractions. The S2 fraction was then centrifuged on a discontinuous sucrose gradient to separate cytosol (S3), crude vesicles and the plasma membrane (P3). Subsequent western blot analysis suggests that dSNAP-29 is equally enriched in the vesicle and the plasma membrane fractions (Fig. 3B). The fractionation profile of dSNAP-29 is more similar to that of n-Syb, a vesicle SNARE, but in contrast to those of syntaxin1 and SNAP-25, which have demonstrated a preference for plasma membrane. Importantly, both dNSF2 and dSNAP appear to cofractionate with dSNAP-29, implicating the two proteins as potential dSNAP-29 regulators on both the vesicles and the plasma membrane.

### dSNAP-29 interacts with dsyntaxin1 and dsyntaxin16

**Figure 4:**
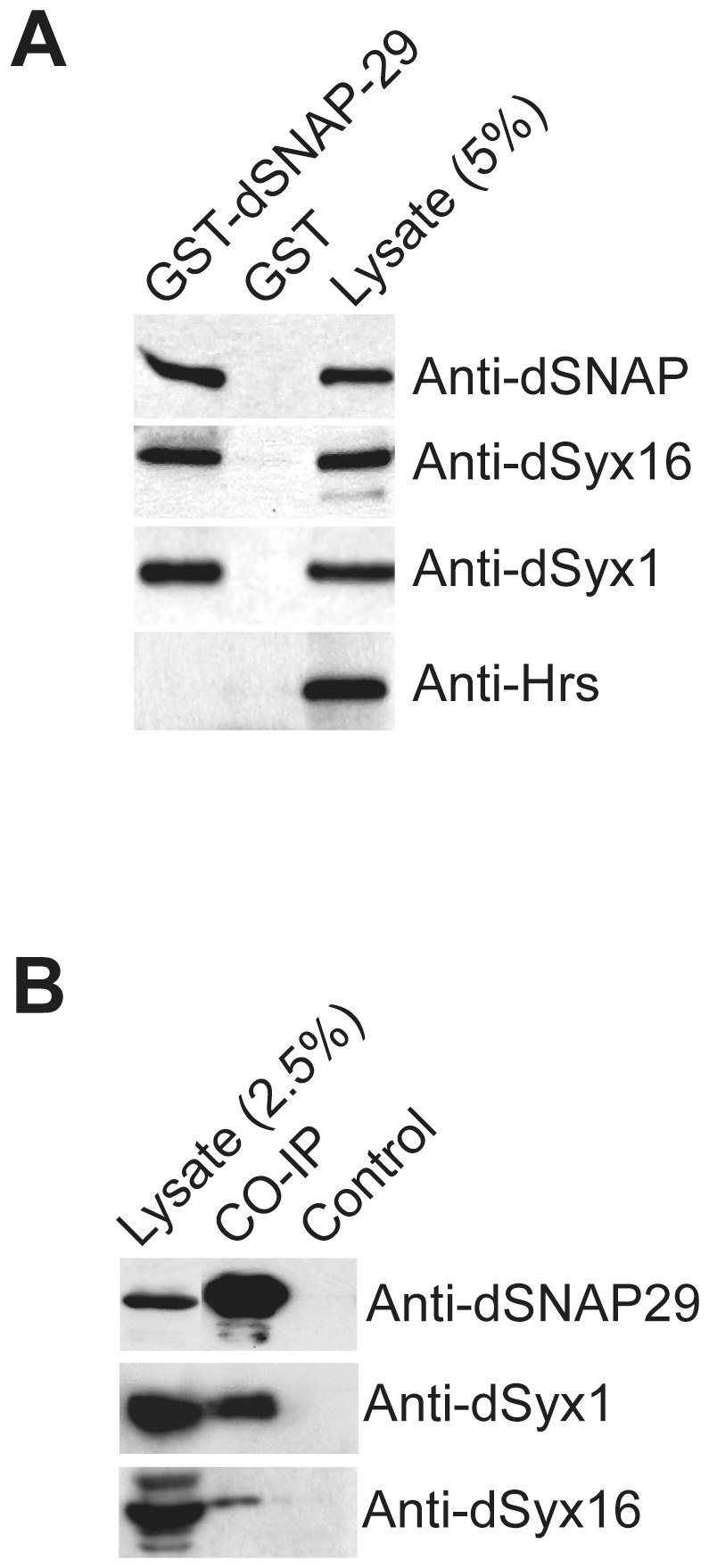
dSNAP-29 interacts with dSyx1 and dSyx16 but not Hrs. A) Immobilized GST-dSNAP29 and GST were incubated with S2 cell lysate. Following extensive washes, Proteins were eluted and subject to SDS-PAGE and western blotting analysis. B) *Drosophila* head lysate was incubated with anti-dSNAP29 crosslinked to protein A beads. Pre-immune serum was used as control.

Previous studies suggest that the mammalian SNAP-29 associates with syntaxins on multiple membranes and may thereby participate in multiple trafficking events [21], [23]. To identify the cognate SNARE partners for the *Drosophila* SNAP-29, we performed a pull-down assay using fresh lysates of cultured Schneider cells (S2). S2 cells are derived from embryonic cells and appear to express dNSF2, dSNAP, and various SNAREs such as dSNAP-29, dSyx1, dSyx 5, and dSyx 16. Using immobilized GST-SNAP-29, we were able to recover about 7% of the SNAP from the lysate (Fig. 4A). Importantly, the same amount of GST did not recover any detectable amount of dSNAP, suggesting that the assay is both specific and efficient. In the meantime, GST-SNAP-29, but not GST, recruited 6% of dsyntaxin1 and 4% of dsyntaxin 16 from the lysate.

To determine whether the interactions exist in vivo, we performed co-immunoprecipitation experiments using overnight embryonic collections. Instead of the peptide antibody, which may not recognize dSNAP-29 in the SNARE complex, we employed affinity-purified serum raised against the full-length recombinant dSNAP-29. The antibody, but not the pre-immune serum, brought down significant amounts of dSNAP-29 from the embryonic lysate, together with dsyntaxin1 and a small amount of dsyntaxin 16 (Fig. 4B). Thus, our studies suggest that dSNAP-29 may serve on the Golgi or TGN as a partner for dsyntaxin16 [41]. It is intriguing however, to learn that dSNAP-29 may actually interact with syntaxin1, considering their very different distribution patterns in the salivary gland (syntaxin1 is on the apical membrane whereas SNAP-29 is localized to the basolateral membrane and the cytoplasm). Further analysis is warranted to determine the physiological relevance of the biochemical interactions

### dSNAP-29 does not form SDS-resistant SNARE complexes

**Figure 5:**
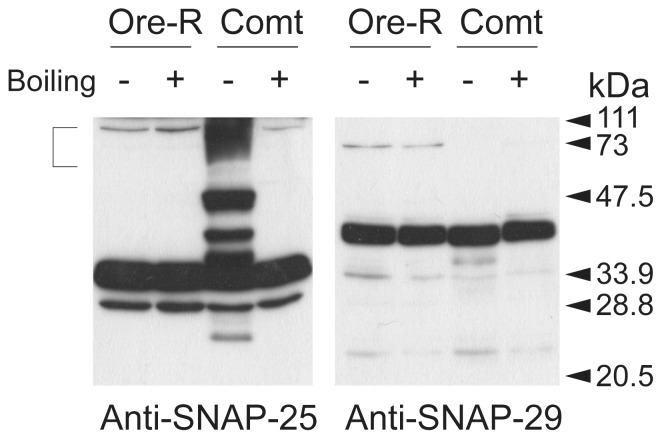
dSNAP-29 does not form SDS-resistant complexes. Heads of heat-challenged *Oregon R* and *Comt^Tp7^* flies were collected in SDS-containing homogenization buffer. Half of each sample was boiled while the other half was not to preserve SNARE complexes. Boiling-sensitive yet SDS-resistant SNARE complexes are indicated by ([).

Some SNAREs readily form stable SDS-resistant complexes whose dissassembly requires the actions of NSF and SNAP. In *Drosophila*, blocking the ATPase activity of NSF-1 by incubation of *comt^TP7^* (a temperature-sensitive allele of *dNSF-1*) adult flies at non-permissive temperatures led to a significant increase of SDS-resistant SNARE complexes detectable by western blot analysis [42] Such complexes appear to contain neuronal SNAREs such as dsyntaxin1 and dSNAP-25, which is in agreement with the notion that dNSF1 is the predominant functional isoform in the adult nervous system [52]. Niemeyer and Schwarz demonstrated that recombinant dSNAP-24 can also form SDS-resistant complexes in vitro [51]. To examine whether dSNAP-29 forms SDS-resistant complex with its cognate SNAREs in vivo, we heat shocked *comt^TP7^* or wild-type adult flies at 37 °C for 10 min, after which the heads were collected and homogenized directly in 1% SDS lysis buffer. Half of the amount of each sample was boiled and the other half was not in order to preserve the complexes. As revealed in Fig. 5, dSNAP-25 from heat-challenged *comt^TP7^* flies accumulated in the SDS-resistant complexes that are disintegrated by boiling. In contrast, dSNAP-29 was not detected in such SDS-resistant complexes when dNSF1 was impaired (Fig. 5). At least three scenarios could account for this. In the first scenario, dSNAP-29 forms SDS-resistant complexes that are sensitive to dNSF-2 rather than to dNSF-1. However, blocking dNSF-2 function in salivary gland cells does not result in any detectable SDS-resistant complex at all (data not shown). In the second scenario, the SNARE complex formed by dSNAP-29 is sensitive to SDS treatment. This is not utterly surprising as most SDS-resistant SNARE complexes reported thus far involve syntaxin1 and are likely responsible for exocytosis. In the third scenario, a very small quantity of dSNAP-29 participates in SDS-resistant complex at a level that is too low to detect. Whether our finding represents a unique case for SNAP-29 in *Drosophila* or might apply to other SNARE complexes remains to be determined.

### dSNAP-29 is a partner of dEHD1 but not Hrs

**Figure 6:**
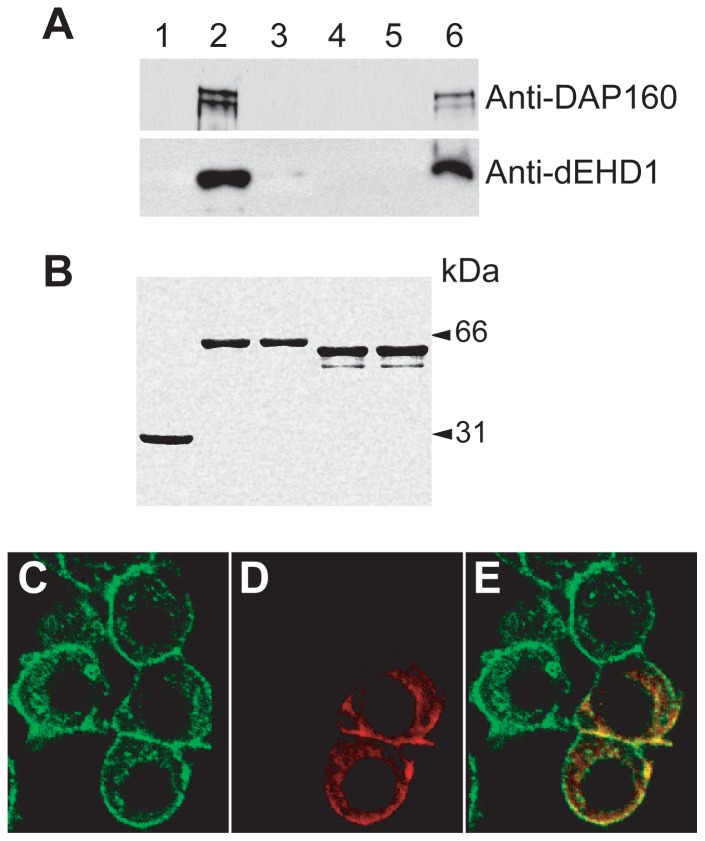
dSNAP-29 selectively interact and partially co-localize with dEHD1 in S2 cells. A) S2 cell lysate (lanes 1, 2, 4) or lysate buffer alone (lanes 3 and 5) was incubated with immobilized GST (lane 1), GST-dSNAP29 (lanes 2 and 3), GST-dSNAP25 (lanes 4 and 5). Associated proteins were separated by SDS-PAGE and blotted by anti-dEHD1. 5% of the lysate used in each pull-down experiment were loaded in lane 6. Shown in B) is a Coomassie-blue stained gel to demonstrate that equal amounts of GST chimeras were used for the pull-down assay. To examine the distribution of dSNAP-29 and dEHD1, S2 cells were transfected with myc-dSNAP29, fixed with methanol, and stained with C) mouse anti-myc and D) rabbit anti-dEHD1. Merged image is shown in E.

The functions of SNAREs are often regulated through reversible interactions with SNARE modulators. Amino-acid sequence analysis revealed a N-terminal NPF motif in dSNAP-29, suggesting that it may interact with EH domain-containing proteins. In the fly genome, there are at least 4 EH domain-containing proteins: PAST1/EHD1, DAP160/intersectin, CG16932/Eps15 and CG6192 [53]. In this study, we tested whether dSNAP-29 interacts with PAST1/EHD1 and DAP160/intersectin, the two *Drosophila* EH domain-containing proteins that we had antibodies for. GST-dSNAP-29, GST-dSNAP-25, and GST were immobilized separately onto glutathione-conjugated agarose beads and then incubated either with homogenized S2 sample or homogenization buffer alone. Subsequent western blot analysis (Fig. 6A) revealed a minimal cross-reaction between affinity purified anti-dEHD1 and recombinant GST-dSNAP29 (lane 3), which runs with the same mobility as the endogenous EHD1. Nonetheless, GST-dSNAP-29 pulled down a significant amount of dEHD1 (12%) and DAP160 (15%) from the lysate (compare lanes 2 and 3) whereas GST (lane 1) or GST-dSNAP25 (lane 4) did not at all, suggesting the interaction between dSNAP-29 and dEHD1 is highly selective. To examine their distribution in vivo, S2 cells transfected with myc-dSNAP29 was immuno-stained with mouse anti-myc (Fig. 6C) and rabbit anti-dEHD1 (Fig. 6D). Significant colocalization was observed on cell surface and on punctate intracellular structures (Fig. 6E). Our finding is line with a recent report which suggests the association of mammalian SNAP-29 with both insulin-like growth factor 1 receptor and EHD1 [39].

### Modulation of dSNAP-29 levels in cultured cells and transgenic flies

To evaluate the functional involvement of dSNAP-29 in membrane traffic, we took two commonly used approaches. The first is to ectopically overexpress the wild-type or the truncated form of dSNAP-29 in transgenic flies. High levels of wild-type dSNAP-29 may sequester its potential binding partners (e.g., dEHD1), causing an inhibition of the putative trafficking step. As a matter of fact, the ubiquitous overexpression of dSNAP-29 controlled by da-GAL4 or actin5-GAL4 led to lethality at the 1^st^ larval stage (Table 1). However, we did not observe any defect in embryogenesis. It has also been demonstrated that a SNAP-25 lacking the C-terminus was able to form complexes with its cognate SNARE partners but failed to execute membrane fusion [10], [54]. Therefore, we constructed UAS-dSNAP-29ΔC, which carries a partial cDNA that does not encode the C-terminal 25 amino acids. Similar to the wild type, overexpression of dSNAP-29ΔC using actin5-GAL4 or da-GAL4 driver is lethal (Table 1). Ectopic expression of dSNAP-29ΔC using C96-GAL4 (wing margin) causes a notch-wing phenotype whereas expression with elav-GAL4 (the nervous system) causes defects in wing extension and development. However, these non-lethal phenotypes were only observed with one of the two UAS- dSNAP-29ΔC lines generated (Table 1).

**Table 1:** Phenotypes caused by ectopic expression of various forms of dSNAP-29.

Gal4 lines	Expression pattern	UAS-dSNAP29	UAS-dSNAP29ΔC	UAS-dSNAP29^RNAi^
actin5-Gal4	ubiquitous	1^st^ instar lethal	lethal	pupal lethal at 29 °C
da-Gal4	ubiquitous	1^st^ instar lethal	lethal	n.d.
C96-Gal4	wing margin	none	Notched wings*	none
24B-Gal4	muscle (late)	none	n.d.	pupal lethal at 29 °C
mhc-Gal4	muscle (early)	none	n.d.	none
elav-Gal4	CNS	none	Abnormal wings*	none

* Not observed in all the independent UAS lines that were tested.

For each UAS construct, several independent lines were tested to rule out positional effect. Visible phenotypes or sterility were scored. The crosses are set at both room temperature and 29 °C unless indicated otherwise. n.d., not determined.

An alternative but more direct approach is to knock down the protein expression by RNA interference. We constructed a UAS-dSNAP-29^RNAi^ transgene to express double-stranded RNA in defined tissues in the fly. When the construct was expressed under the control of the ubiquitous actin5-GAL4 driver at room temperature, we achieved excellent reduction of the protein level (around 95%; Fig. 1D). Although flies appear normal under these conditions, incubation at 29 °C causes lethality at the pupal stage (Table 1). Lethality was also observed when the 24B-GAL4 driver (expressed mainly in the muscle) was used, whereas the elav-GAL4 driver (expressed mainly in the nervous system) does not produce any visible phenotype. Thus, it appears that dSNAP-29 may play an essential role in the post synaptic cells although it may also be actively involved in other cells/tissues.

## Discussion

In multicellular systems, SNAP-25/23 functions predominantly on the plasma membrane, whereas SNAP-29 is found mainly in the cytoplasm. An important difference between SNAP-25 and SNAP-29 is that the later possesses an N-terminal NPF motif, which allows for interaction with EH domain-containing proteins. In our study, dSNAP-29 has been shown to interact with dEHD1 and DAP160, two of the four known EH domain-containing proteins in the fly genome [53]. Whereas *Drosophila* EHD1 has been implicated in endocytosis [55], its worm orthologue RME-1 and its mammalian orthologue EHD1 have been implicated in the recycling of a set of endocytosed molecules back to the plasma membrane [56], [57], [58]. DAP160 on the other hand, may act as a scaffold protein by interacting with the endocytic machinery including dynamin, clathrin and synaptojanin [59]. Intriguingly, SNAP-29, EHD1, and DAP160 do not appear to be present in the yeast genome, suggesting that they are required for a more specialized pathway that has evolved only in multicellular organisms.

In an attempt to address the physiological relevance of the interactions between dSNAP-29 and the EH domain-containing proteins, we transfected S2 cells with the myc-dSNAP-29 construct and performed immunostaining using anti-dEHD1 and anti-myc antibodies. At low expression levels, myc-SNAP-29 appears to partially colocalize with dEHD1 at the plasma membrane as well as the peripheral regions underneath the plasma membrane (Fig. 6 C,D,E). However, the plasma-membrane distribution of dSNAP-29 was not observed when cells were stained with anti-dSNAP-29. Which staining pattern more accurately represents the in vivo distribution of the protein is not clear.

How do we explain the interaction between dSNAP-29 and the syntaxins? It may be counter-intuitive to suggest that dSyx1 and dSNAP-29 can actually function together in the salivary gland. dSyx1 is found on the apical membrane whereas dSNAP-29 is not. Since dSNAP-25 is not expressed in the salivary gland, a likely partner for syntaxin 1 during granule secretion is probably dSNAP-24, which is localized to the secretory granules and which redistributes upon exocytosis. dSNAP-29 is not enriched on those secretory granules, but is found in the remaining cytoplasm, and may very well be on recycling vesicles. In cultured S2 cells, transfected dSNAP-29 appears to partially colocalize with dSyx1 on intracellular punctae, with a pattern differing significantly from that of ER, Golgi, endosomes, or lysosomes (data not shown). It is possible that the two proteins interact only transiently during the recycling pathway. As well, we cannot rule out the possibility that dSNAP-29 may adopt different distribution patterns in different tissues.

The demonstration that dSNAP-29 also interacts with dsyntaxin 16 does not necessarily imply that dSNAP-29 is promiscuous. In a separate study, we showed that dsyntaxin 5 does not interact with dSNAP-29 in vitro (Hsien and Trimble, unpublished), which is in agreement with reports on its mammalian orthologue. However, unlike dsyntaxin 5, which is predominantly on the cis-Golgi, dsyntaxin 16 is localized to a late Golgi compartment, possibly in the trans-Golgi network. More importantly, mammalian syntaxin 16 has recently been implicated in retrograde transport from the endosomes [60], consistent with the notion that SNAP-29 is also involved in the recycling pathway. Recently, dSNAP-29 has been shown to regulate autophagy along with dsynxtin11 and dVAMP7 [30], arguing for a more versatile role for SNAP-29 in intracellular membrane traffic.

## References

[1] Chen YA, Scheller RH (2001) SNARE-mediated membrane fusion. Nat Rev Mol Cell Biol 2: 98–106. 11252968 10.1038/35052017

[2] Jahn R, Sudhof TC (1999) Membrane fusion and exocytosis. Annu Rev Biochem 68: 863–911. 10872468 10.1146/annurev.biochem.68.1.863

[3] Rothman JE (1994) Intracellular membrane fusion. Adv Second Messenger Phosphoprotein Res 29: 81–96. 7848733 10.1016/s1040-7952(06)80008-x

[4] Pelham HR (2001) SNAREs and the specificity of membrane fusion. Trends Cell Biol 11: 99–101. 11306253 10.1016/s0962-8924(01)01929-8

[5] Bock JB, Matern HT, Peden AA, Scheller RH (2001) A genomic perspective on membrane compartment organization. Nature 409: 839–841. 11237004 10.1038/35057024

[6] Holt M, Varoqueaux F, Wiederhold K, Takamori S, Urlaub H, et al (2006) Identification of SNAP-47, a novel Qbc-SNARE with ubiquitous expression. J Biol Chem 281: 17076–17083. 16621800 10.1074/jbc.M513838200

[7] Oyler GA, Higgins GA, Hart RA, Battenberg E, Billingsley M, et al (1989) The identification of a novel synaptosomal-associated protein, SNAP-25, differentially expressed by neuronal subpopulations. J Cell Biol 109: 3039–3052. 2592413 10.1083/jcb.109.6.3039 PMC2115928

[8] Kawasaki F, Ordway RW (2009) Molecular mechanisms determining conserved properties of short-term synaptic depression revealed in NSF and SNAP-25 conditional mutants. Proc Natl Acad Sci U S A 106: 14658–14663. 19706552 10.1073/pnas.0907144106 PMC2732793

[9] Blasi J, Chapman ER, Link E, Binz T, Yamasaki S, et al (1993) Botulinum neurotoxin A selectively cleaves the synaptic protein SNAP-25. Nature 365: 160–163. 8103915 10.1038/365160a0

[10] Wei S, Xu T, Ashery U, Kollewe A, Matti U, et al (2000) Exocytotic mechanism studied by truncated and zero layer mutants of the C-terminus of SNAP-25. Embo J 19: 1279–1289. 10716928 10.1093/emboj/19.6.1279 PMC305669

[11] Ravichandran V, Chawla A, Roche PA (1996) Identification of a novel syntaxin- and synaptobrevin/VAMP-binding protein, SNAP-23, expressed in non-neuronal tissues. J Biol Chem 271: 13300–13303. 8663154 10.1074/jbc.271.23.13300

[12] Wang G, Witkin JW, Hao G, Bankaitis VA, Scherer PE, et al (1997) Syndet is a novel SNAP-25 related protein expressed in many tissues. J Cell Sci 110 (Pt 4): 505–513. 10.1242/jcs.110.4.505 9067602

[13] Foster LJ, Yaworsky K, Trimble WS, Klip A (1999) SNAP23 promotes insulin-dependent glucose uptake in 3T3-L1 adipocytes: possible interaction with cytoskeleton. Am J Physiol 276: C1108–1114. 10329959 10.1152/ajpcell.1999.276.5.C1108

[14] Chen D, Lemons PP, Schraw T, Whiteheart SW (2000) Molecular mechanisms of platelet exocytosis: role of SNAP-23 and syntaxin 2 and 4 in lysosome release. Blood 96: 1782–1788. 10961877

[15] Galli T, Zahraoui A, Vaidyanathan VV, Raposo G, Tian JM, et al (1998) A novel tetanus neurotoxin-insensitive vesicle-associated membrane protein in SNARE complexes of the apical plasma membrane of epithelial cells. Mol Biol Cell 9: 1437–1448. 9614185 10.1091/mbc.9.6.1437 PMC25366

[16] Leung SM, Chen D, DasGupta BR, Whiteheart SW, Apodaca G (1998) SNAP-23 requirement for transferrin recycling in Streptolysin-O-permeabilized Madin-Darby canine kidney cells. J Biol Chem 273: 17732–17741. 9651373 10.1074/jbc.273.28.17732

[17] Low SH, Chapin SJ, Wimmer C, Whiteheart SW, Komuves LG, et al (1998) The SNARE machinery is involved in apical plasma membrane trafficking in MDCK cells. J Cell Biol 141: 1503–1513. 9647644 10.1083/jcb.141.7.1503 PMC2133007

[18] Lafont F, Verkade P, Galli T, Wimmer C, Louvard D, et al (1999) Raft association of SNAP receptors acting in apical trafficking in Madin-Darby canine kidney cells. Proc Natl Acad Sci U S A 96: 3734–3738. 10097106 10.1073/pnas.96.7.3734 PMC22363

[19] Vilinsky I, Stewart BA, Drummond J, Robinson I, Deitcher DL (2002) A Drosophila SNAP-25 null mutant reveals context-dependent redundancy with SNAP-24 in neurotransmission. Genetics 162: 259–271. 12242238 10.1093/genetics/162.1.259 PMC1462260

[20] Jurado S, Goswami D, Zhang Y, Molina AJ, Sudhof TC, et al (2013) LTP requires a unique postsynaptic SNARE fusion machinery. Neuron 77: 542–558. 23395379 10.1016/j.neuron.2012.11.029 PMC3569727

[21] Steegmaier M, Yang B, Yoo JS, Huang B, Shen M, et al (1998) Three novel proteins of the syntaxin/SNAP-25 family. J Biol Chem 273: 34171–34179. 9852078 10.1074/jbc.273.51.34171

[22] Wong SH, Xu Y, Zhang T, Griffiths G, Lowe SL, et al (1999) GS32, a novel Golgi SNARE of 32 kDa, interacts preferentially with syntaxin 6. Mol Biol Cell 10: 119–134. 9880331 10.1091/mbc.10.1.119 PMC25158

[23] Hohenstein AC, Roche PA (2001) SNAP-29 is a promiscuous syntaxin-binding SNARE. Biochem Biophys Res Commun 285: 167–171. 11444821 10.1006/bbrc.2001.5141

[24] Wesolowski J, Caldwell V, Paumet F (2012) A novel function for SNAP29 (synaptosomal-associated protein of 29 kDa) in mast cell phagocytosis. PLoS One 7: e49886. 23185475 10.1371/journal.pone.0049886 PMC3503860

[25] Rapaport D, Lugassy Y, Sprecher E, Horowitz M (2010) Loss of SNAP29 impairs endocytic recycling and cell motility. PLoS One 5: e9759. 20305790 10.1371/journal.pone.0009759 PMC2841205

[26] Sato M, Saegusa K, Sato K, Hara T, Harada A (2011) Caenorhabditis elegans SNAP-29 is required for organellar integrity of the endomembrane system and general exocytosis in intestinal epithelial cells. Mol Biol Cell 22: 2579–2587. 21613542 10.1091/mbc.E11-04-0279 PMC3135482

[27] Kang J, Bai Z, Zegarek MH, Grant BD, Lee J (2011) Essential roles of snap-29 in C. elegans. Dev Biol 355: 77–88. 21545795 10.1016/j.ydbio.2011.04.013 PMC3118655

[28] Su Q, Mochida S, Tian JH, Mehta R, Sheng ZH (2001) SNAP-29: a general SNARE protein that inhibits SNARE disassembly and is implicated in synaptic transmission. Proc Natl Acad Sci U S A 98: 14038–14043. 11707603 10.1073/pnas.251532398 PMC61163

[29] Hamasaki M, Furuta N, Matsuda A, Nezu A, Yamamoto A, et al (2013) Autophagosomes form at ER-mitochondria contact sites. Nature 495: 389–393. 23455425 10.1038/nature11910

[30] Takats S, Nagy P, Varga A, Pircs K, Karpati M, et al (2013) Autophagosomal Syntaxin17-dependent lysosomal degradation maintains neuronal function in Drosophila. J Cell Biol 201: 531–539. 23671310 10.1083/jcb.201211160 PMC3653357

[31] Chin LS, Nugent RD, Raynor MC, Vavalle JP, Li L (2000) SNIP, a novel SNAP-25-interacting protein implicated in regulated exocytosis. J Biol Chem 275: 1191–1200. 10625663 10.1074/jbc.275.2.1191

[32] Komada M, Soriano P (1999) Hrs, a FYVE finger protein localized to early endosomes, is implicated in vesicular traffic and required for ventral folding morphogenesis. Genes Dev 13: 1475–1485. 10364163 10.1101/gad.13.11.1475 PMC316760

[33] Lloyd TE, Atkinson R, Wu MN, Zhou Y, Pennetta G, et al (2002) Hrs regulates endosome membrane invagination and tyrosine kinase receptor signaling in Drosophila. Cell 108: 261–269. 11832215 10.1016/s0092-8674(02)00611-6

[34] Shih SC, Katzmann DJ, Schnell JD, Sutanto M, Emr SD, et al (2002) Epsins and Vps27p/Hrs contain ubiquitin-binding domains that function in receptor endocytosis. Nat Cell Biol 4: 389–393. 11988742 10.1038/ncb790

[35] Raiborg C, Bache KG, Gillooly DJ, Madshus IH, Stang E, et al (2002) Hrs sorts ubiquitinated proteins into clathrin-coated microdomains of early endosomes. Nat Cell Biol 4: 394–398. 11988743 10.1038/ncb791

[36] Kwong J, Roundabush FL, Hutton Moore P, Montague M, Oldham W, et al (2000) Hrs interacts with SNAP-25 and regulates Ca(2+)-dependent exocytosis. J Cell Sci 113 (Pt 12): 2273–2284. 10.1242/jcs.113.12.2273 10825299

[37] Bean AJ, Seifert R, Chen YA, Sacks R, Scheller RH (1997) Hrs-2 is an ATPase implicated in calcium-regulated secretion. Nature 385: 826–829. 9039916 10.1038/385826a0

[38] Okamoto M, Schoch S, Sudhof TC (1999) EHSH1/intersectin, a protein that contains EH and SH3 domains and binds to dynamin and SNAP-25. A protein connection between exocytosis and endocytosis? J Biol Chem 274: 18446–18454. 10373452 10.1074/jbc.274.26.18446

[39] Rotem-Yehudar R, Galperin E, Horowitz M (2001) Association of insulin-like growth factor 1 receptor with EHD1 and SNAP29. J Biol Chem 276: 33054–33060. 11423532 10.1074/jbc.M009913200

[40] Xu H, Brill JA, Hsien J, McBride R, Boulianne GL, et al (2002) Syntaxin 5 is required for cytokinesis and spermatid differentiation in Drosophila. Dev Biol 251: 294–306. 12435359 10.1006/dbio.2002.0830

[41] Xu H, Boulianne GL, Trimble WS (2002) Drosophila syntaxin 16 is a Q-SNARE implicated in Golgi dynamics. J Cell Sci 115: 4447–4455. 12414991 10.1242/jcs.00139

[42] Mohtashami M, Stewart BA, Boulianne GL, Trimble WS (2001) Analysis of the mutant Drosophila N-ethylmaleimide sensitive fusion-1 protein in comatose reveals molecular correlates of the behavioural paralysis. J Neurochem 77: 1407–1417. 11389191 10.1046/j.1471-4159.2001.00363.x

[43] Clemens JC, Worby CA, Simonson-Leff N, Muda M, Maehama T, et al (2000) Use of double-stranded RNA interference in Drosophila cell lines to dissect signal transduction pathways. Proc Natl Acad Sci U S A 97: 6499–6503. 10823906 10.1073/pnas.110149597 PMC18635

[44] Brand AH, Perrimon N (1993) Targeted gene expression as a means of altering cell fates and generating dominant phenotypes. Development 118: 401–415. 8223268 10.1242/dev.118.2.401

[45] Lindsley D, Zimm GG (1992) The Genome of Drosophila melanogaster. San Diego: Academic Press.

[46] Rubin GM, Spradling AC (1982) Genetic transformation of Drosophila with transposable element vectors. Science 218: 348–353. 6289436 10.1126/science.6289436

[47] Gonzalo S, Greentree WK, Linder ME (1999) SNAP-25 is targeted to the plasma membrane through a novel membrane-binding domain. J Biol Chem 274: 21313–21318. 10409690 10.1074/jbc.274.30.21313

[48] Gonzalo S, Linder ME (1998) SNAP-25 palmitoylation and plasma membrane targeting require a functional secretory pathway. Mol Biol Cell 9: 585–597. 9487128 10.1091/mbc.9.3.585 PMC25287

[49] Low SH, Roche PA, Anderson HA, van Ijzendoorn SC, Zhang M, et al (1998) Targeting of SNAP-23 and SNAP-25 in polarized epithelial cells. J Biol Chem 273: 3422–3430. 9452464 10.1074/jbc.273.6.3422

[50] Santolini E, Salcini AE, Kay BK, Yamabhai M, Di Fiore PP (1999) The EH network. Exp Cell Res 253: 186–209. 10579923 10.1006/excr.1999.4694

[51] Niemeyer BA, Schwarz TL (2000) SNAP-24, a Drosophila SNAP-25 homologue on granule membranes, is a putative mediator of secretion and granule-granule fusion in salivary glands. J Cell Sci 113 (Pt 22): 4055–4064. 10.1242/jcs.113.22.4055 11058092

[52] Stewart BA, Mohtashami M, Rivlin P, Deitcher DL, Trimble WS, et al (2002) Dominant-negative NSF2 disrupts the structure and function of Drosophila neuromuscular synapses. J Neurobiol 51: 261–271. 12150502 10.1002/neu.10059

[53] Confalonieri S, Di Fiore PP (2002) The Eps15 homology (EH) domain. FEBS Lett 513: 24–29. 11911876 10.1016/s0014-5793(01)03241-0

[54] Chen YA, Scales SJ, Patel SM, Doung YC, Scheller RH (1999) SNARE complex formation is triggered by Ca2+ and drives membrane fusion. Cell 97: 165–174. 10219238 10.1016/s0092-8674(00)80727-8

[55] Olswang-Kutz Y, Gertel Y, Benjamin S, Sela O, Pekar O, et al (2009) Drosophila Past1 is involved in endocytosis and is required for germline development and survival of the adult fly. J Cell Sci 122: 471–480. 19174465 10.1242/jcs.038521

[56] Caplan S, Naslavsky N, Hartnell LM, Lodge R, Polishchuk RS, et al (2002) A tubular EHD1-containing compartment involved in the recycling of major histocompatibility complex class I molecules to the plasma membrane. Embo J 21: 2557–2567. 12032069 10.1093/emboj/21.11.2557 PMC126039

[57] Grant B, Zhang Y, Paupard MC, Lin SX, Hall DH, et al (2001) Evidence that RME-1, a conserved C. elegans EH-domain protein, functions in endocytic recycling. Nat Cell Biol 3: 573–579. 11389442 10.1038/35078549

[58] Lin SX, Grant B, Hirsh D, Maxfield FR (2001) Rme-1 regulates the distribution and function of the endocytic recycling compartment in mammalian cells. Nat Cell Biol 3: 567–572. 11389441 10.1038/35078543

[59] Roos J, Kelly RB (1998) Dap160, a neural-specific Eps15 homology and multiple SH3 domain-containing protein that interacts with Drosophila dynamin. J Biol Chem 273: 19108–19119. 9668096 10.1074/jbc.273.30.19108

[60] Mallard F, Tang BL, Galli T, Tenza D, Saint-Pol A, et al (2002) Early/recycling endosomes-to-TGN transport involves two SNARE complexes and a Rab6 isoform. J Cell Biol 156: 653–664. 11839770 10.1083/jcb.200110081 PMC2174079

